# Supplementary material for: Are carbon emissions trading and green financial instruments synergistic? -Comprehensive quantitative research based on content analysis
Source: PLoS One. 2024 Mar 7;19(3):e0298601. doi: 10.1371/journal.pone.0298601 (PMC10919601; doi:10.1371/journal.pone.0298601)
Supplement: S1 Data — (ZIP) [file pone.0298601.s001.zip › Data/地级市Z_F工作报告.zip!/─┌├╔╣┼/║⌠║═║╞╠╪╩╨/└┤╫╘╣1⁄2╓┌║┼í╛┬φ┐╦╩2╛▌═°í┐_2016.docx]

各位代表：

现在，我代表市人民政府向大会报告工作，请连同《呼和浩特市国民经济和社会发展第十三个五年规划纲要(草案)》一并审议，并请市政协委员和列席会议的同志们提出意见。

一、“十二五”时期和2015年经济社会发展回顾

“十二五”以来，市人民政府深入贯彻落实党的十八大和十八届三中、四中、五中全会精神，深入贯彻落实习近平总书记系列重要讲话和考察内蒙古重要讲话精神，深入贯彻落实自治区“8337”发展思路以及建设“五大基地”、打造“六道亮丽风景线”等决策部署，按照市委“打造两个一流、推进三个建设、实现两个率先”总体要求，主动认识新常态、适应新常态、引领新常态，着力稳增长、促改革、调结构、惠民生、防风险，经济社会发展实现重大跨越。

——经济实力显著增强。2010年到2015年，地区生产总值由1865.7亿元增加到3090.5亿元，年均增长9.7%，跨越2000亿元和3000亿元两个台阶。人均GDP由1万美元提高到1.6万美元，位居全国大中城市前列。一般公共预算收入由126.8亿元增加到247.4亿元，年均增长18%，高于GDP增速8.3个百分点。社会消费品零售总额达到1353.5亿元，比2010年翻了近一番，年均增长12.3%。五年累计完成固定资产投资7193亿元，是前11个五年计划投资总量的1.5倍，年均增长17.9%。全面建成小康社会实现程度达到93%。成功入选国家创新型试点城市、国家服务业综合改革试点城市、国家电子商务示范城市、国家新能源示范城市、国家智慧城市试点、“宽带中国”示范城市、全国文明城市提名城市。

——产业结构明显优化。三次产业结构由4.9∶36.4∶58.7演进为4.1∶28.0∶67.9，形成了以服务业为主导，多元发展、多极支撑的产业格局。都市型现代农业加快发展，本地蔬菜供应率由36%提高到57.8%，奶牛规模化养殖水平由50%提高到97%，农业产业化水平居全区首位。“五大基地”建设取得重要进展，培育形成了绿色农畜产品加工、清洁能源、光伏材料、生物制药等一批在全国具有明显优势的特色产业集群。伊利、蒙牛两大企业主营业务收入合计超过1000亿元，电力总装机超过1000万千瓦，单晶硅和多晶硅产量分别达到8569吨和4966吨，生物发酵能力达到10万吨，战略性新兴产业占全市工业比重超过30%。新兴服务业蓬勃发展，建成全国最大的云数据基地，各类电商企业达到1600多家，旅游总收入、接待总人数稳居全区首位，自治区级服务业集聚区达到16个，新兴服务业占第三产业比重超过40%。

——城乡面貌发生深刻变化。按照“改造提升中心城区，规划建设东部新区，打造北部生态观光带，建设南部现代产业园”的城市发展思路，加快一流首府城市建设。启动实施了大青山前坡生态保护综合治理、六大出城口及京藏高速两侧绿化整治、“引黄入呼”二期、“气化呼和浩特”、市政道路桥梁管网等一批重点工程，城市发展步入了由外延扩张式向内涵提升式转变的新阶段。建成区面积由2010年的174平方公里扩展到2015年的260平方公里，常住人口城镇化率由62.5%提高到67.5%，市政道路面积从1848万平方米增加到2654万平方米，绿化覆盖面积从9.3万亩增加到14.4万亩，雨污水和供水管网从1900公里增加到2705公里，污水集中处理率从77.1%提高到91.8%，燃气普及率由92.4%提高到98.6%，城市综合承载力和宜居水平显著提升。各旗县城镇持续扩容提质，公共服务能力明显增强。

——发展保障水平全面提升。建成高速公路5条、一级公路7条，公路总里程由6284公里增加到7295公里，路网密度达到42.4公里/百平方公里，居全区首位。完成货运周转量1613.6亿吨公里，比“十一五”增加86%。开工建设呼张高铁、呼准鄂城际铁路，开通运行呼包集城际动车组，结束了自治区没有动车组的历史。国内外通航城市达到64个，运营航线139条，旅客吞吐量2983万人次，比“十一五”增长2倍。新建扩建110千伏及以上等级变电站33座，新增供电线路1054公里，电网架构进一步优化。完成23座病险水库除险加固，对134万亩灌区进行了节水改造，新增有效灌溉面积137万亩。完成林业生态建设208.8万亩，全市森林覆盖率由22.4%提高到24.9%，生态环境持续改善。

——民生社会事业协调进步。五年累计投入民生领域资金935亿元，占一般公共预算支出的63%以上。城乡常住居民人均可支配收入年均分别增长10.6%和11.5%，达到37362元和13491元。城镇登记失业率始终控制在4%以内。覆盖城乡和各类人群的社会保障体系更加健全，企业退休职工、城乡居民人均养老金分别提高53.1%、94.6%，城市最低工资标准、城镇和农村低保标准分别提高82%、66.2%和91.8%。连续五年提高环卫工人待遇，累计增幅130%。“三个一”民生实事惠及24万农户、5.1万名贫困家庭大学生和467户零就业家庭。新建、改扩建中小学幼儿园131所，完成市第一医院、口腔医院迁建及五个旗县中心医院改扩建等一批医疗卫生项目，“入园难”、“上学难”、“看病难”等问题得到有效缓解。

各位代表，刚刚过去的2015年，我们圆满完成了市十四届人大三次会议确定的各项任务。地区生产总值增长8.3%，一般公共预算收入增长17%，固定资产投资增长15.8%，社会消费品零售总额增长7.8%，城乡常住居民人均可支配收入均增长7.6%，居民消费价格指数上涨1.8%。

主要指标处于合理区间，位居全区各盟市前列，经济运行总体向好，呈现出结构优化、动力转换、后劲增强的显著特点。今年1月6日，国务院常务会议决定对落实重点政策成效较明显的20个地市、20个县区予以通报表扬，并在2016年实行“免督查”和一系列激励措施，我市是全区唯一受到国务院表扬的地级市。在中央电视台联合国家统计局等单位开展的《中国经济生活大调查》评选中，我市成为全国幸福感较高的十个城市之一。由中国社科院发布的2015年《公共服务蓝皮书》中，我市在全国主要城市基本公共服务满意度排行榜上位列第十七，高于北京、济南等城市。一年来，我们主要做了以下工作：

(一)全力稳定经济增长。认真落实各项政策，研究出台加强和规范工业招商引资、加快发展服务业、促进房地产市场平稳健康发展等一系列政策措施，为企业安排和争取产业扶持资金7220万元、电价补贴1.3亿元，协调落实“助保贷”1.5亿元，发行小微企业增信集合债6.5亿元，对稳定经济增长发挥了重要作用。积极扩大有效投资，实施亿元以上项目429个，完成投资908.3亿元。中环光伏三期、晶环蓝宝石二期、华夏聚光一期、大唐托电五期、京能盛乐热电等项目有序推进；庆华煤基清洁能源多联产项目获得“大路条”；沙良和城发恼包、亿阳等大型物流园区投入运营；武川至葛根塔拉一级公路建成通车；引进深圳华强、北大青鸟、西部控股、海润在线等一批文化产业项目。创新投融资机制，搭建了城乡建设投资、青山文化旅游投资、成石基金、呼穗基金等平台，融资到位381亿元。争取自治区地方政府债券199.4亿元、上级专项转移支付72.7亿元。争取国家财政部PPP项目3个，总投资381.5亿元。恒泰证券在香港联交所成功上市，9家企业在“新三板”挂牌，51家企业在内蒙古股权交易中心挂牌。

(二)加快产业转型升级。全力推进新兴服务业加快发展。云计算服务器能力达到66万台，入驻数据应用企业33家。新增各类金融机构12家，金融机构存贷款余额分别增长13.6%和18%，金融业对经济增长的贡献率达到18.5%。全年网络零售交易额实现136亿元，增长76.6%。快递业收入增长10.9%。举办各类展会92场，成交及意向协议总额超过100亿元。接待旅游总人数3057.4万人次，实现旅游总收入508.6亿元，分别增长20.2%和23.1%。文化产业完成增加值93亿元，占GDP比重超过3%。服务业增加值增长8.8%，高于上年0.5个百分点，提供的税收突破210亿元，吸纳了83.5%的城镇新增就业，对GDP的贡献率达到68.7%。大力促进工业转型升级。制定出台《工业转型升级引导办法》，实施亿元以上工业重点项目63项，新增规模以上工业企业23家，新认定国家高新技术企业22家、国家级技术创新示范企业2家。规模以上工业增加值增长10%，增速居全区第二位；工业税金同比增长24.8%，居全区第一位。生物医药、光伏材料等战略性新兴产业实现产值516.5亿元，对工业增长贡献率达到85.4%。大力提升农业生产效益。全市主要农作物良种覆盖率超过96%，农业综合机械化率达到86%，农业科技贡献率提高到61%，建成的温室大棚投产率达到98%，奶牛平均单产提高到7吨，现代农业发展水平显著提升。

(三)不断提升城市建设管理水平。开工建设地铁首开段，机场迁建进入最后审批阶段，历时两年、单体规模和投资最大的城市快速路全线建成通车，极大缓解了城市交通压力，带动了城市快速发展。启动实施拆改建三年行动计划，拆除棚户区、城乡危房235万平方米，综合改造老旧小区300个，对85个老旧小区供排水管网及二次加压设备进行升级改造，打通断头路13条，建成便民市场47个，城市宜居水平进一步提升。开通公交快线2条、微循环线路4条、城乡线路4条，新建公共自行车站点130个，投放公共自行车5040辆，城乡居民出行更加便捷。拆并整合及燃气化改造锅炉133台，关停污染小企业178家，淘汰黄标车7394辆，空气质量在北方15个省会城市中排名第一。完成辛辛板等3座污水处理厂提标改造，排放标准提升至一级A，污水处理达到国内先进水平。新建改建公园和游园6个，新增绿地面积1.55万亩，成功创建为国家园林城市。全面开展地下管网地理信息普查，对50多万户燃气用户和30万只液化气瓶进行安全检查，集中开展渣土扬尘、户外广告、交通秩序等专项整治，解决了一批影响城市形象和安全的老大难问题。

(四)全面深化改革开放。坚持以问题为导向，推进了21项改革试点和187项重点改革任务。取消、下放和调整行政审批项目76项，行政权力事项由9547项减至5429项。完善公共资源交易制度，建设工程和政府采购项目实现网上招投标。加快商事制度改革，市场主体和注册资本分别增长28.2%和22.9%。武川县农村土地承包经营权确权登记颁证试点工作基本完成，和林县全国农村土地征收改革试点工作取得重要进展。教育招生考试改革、基层医疗卫生机构综合改革、公共文化服务标准化均等化、政府购买公共服务等领域改革取得阶段性成果。主动融入国家“一带一路”发展战略，成功举办首届中国-蒙古国博览会，区域影响力和对外开放水平显著提升。

(五)着力保障和改善民生。投入资金71.7亿元，完成509个村的“十个全覆盖”工程，改造危房38806户，硬化街巷2023公里，解决18.6万人的安全饮水问题，农村面貌实现新变化。认定国家小微企业创业示范基地1家、自治区众创空间试点基地8家，城镇新增就业4万人，高校毕业生就业率达到91.7%，城镇登记失业率为3.56%，低于年初3.8%的控制性目标。新增环卫工人1200名，为环卫工人全部办理大额意外伤害商业保险，并定向安排公租房145套。投入资金1.05亿元，妥善安置破产企业职工1825名。新建各类保障性安居房2.9万套，下大力解决了近4万户群众回迁房安置遗留问题，为3.7万户居民解决了房屋产权办证问题，涉及群众切身利益的一大批疑难问题得到妥善解决。

(六)全面推进各项社会事业发展。开工建设儿童探索博物馆，新建、改扩建中小学、幼儿园14所，公开招聘教师622名。设立3000万元文化产业奖励扶持资金，大盛魁文化创意产业园获得国家级文化产业示范基地称号。在全国率先开展新农合跨省就医异地即时结算，新农合人均筹资标准提高到504元，在全区排第三位。全民健身中心体育馆、青城公园体育馆、公主府公园体育馆建成使用，呼和浩特中优足球队获得中甲联赛第6名，我市荣获全国体育事业突出贡献奖。启动运行阳光信访信息系统平台，群众来信来访批次和人次分别下降10.8%和11.7%。深入推进平安首府建设，食品药品安全四级监管网络初步形成，刑事案件发案率下降8.7%，安全生产事故起数、死亡人数分别下降23.6%和0.79%。国防建设、民兵应急力量建设、双拥优抚、外事侨务、民族宗教、人防、气象、防震减灾和工会、共青团、妇女儿童、老龄、慈善、红十字、残疾人等各项事业取得新成绩。

(七)进一步加强政府自身建设。认真践行“三严三实”，严厉整治“四风”积弊，全年公开通报违反中央八项规定精神和损害群众利益的典型问题24批125起，曝光181人，立案433件(次)，给予党政纪处分440人。深入开展“勇于担当、攻坚克难”主题活动，242项攻坚克难任务取得重要进展。全面推进依法行政，建立重大行政决策程序制度和政府法律顾问制度，全年完成地方性法规立法计划7件、政府规章1件，宣布废止和失效文件33件。定期向市人大报告工作，向市政协通报工作，全年办理人大代表意见建议220件、政协提案553件。主动回应社会关切，举办新闻发布会、网络发布会、通报会66场。严格落实党风廉政建设“两个责任”，强化行政监察和审计监督，始终保持了预防和惩治腐败的高压态势。

各位代表，经过全市各族人民的顽强拼搏和持续奋斗，实现了“十二五”圆满收官，呼和浩特的发展站在了一个新的历史起点上。回首“十二五”时期的发展，我们深深感到：这五年是我市综合实力提升最快、城乡面貌变化最大、社会建设成就最好、人民群众得到实惠最多的时期之一。“十二五”时期取得的成绩，主要得益于我们始终与以习近平同志为核心的党中央保持高度一致，坚定不移地贯彻落实党中央国务院、自治区党委政府和市委的决策部署，自觉接受市人大及其常委会的法律监督和工作监督、市政协的民主监督以及社会舆论监督，确保了全市经济社会发展沿着正确的方向行稳致远。得益于我们始终坚持发展第一要务不动摇，主动适应、积极引领经济发展新常态，勇于担当、攻坚克难、真抓实干，牢牢把握了发展的主动权。得益于我们始终把保障和改善民生作为一切工作的出发点和落脚点，以经济发展促进民生改善，充分调动了各方面的积极性主动性创造性，凝聚起了推动发展的强大动力。在此，我代表市人民政府，向全市各族人民，向辛勤工作在各条战线的广大干部群众，向所有关心支持呼和浩特建设发展的朋友们，表示衷心的感谢并致以崇高的敬意!

与此同时，我们也清醒地认识到发展中还存在一些突出问题，面临不少挑战。一是全市整体经济实力还不够强，工农业短板尚未补齐，现代服务业发展不够充分，增长动能转换和产业转型升级仍需加快。二是城市综合承载力和竞争力还不够强，市政基础设施欠账多，城市管理不够精细，农村公共服务体系还不够健全，城乡二元和城区二元、农村二元结构现象交织，推进城乡一体化仍需付出艰辛努力。三是社会建设和管理还存在薄弱环节，部分群众生活还比较困难，在全区率先全面建成小康社会任务艰巨。四是行政审批环节多、效率不高等问题依然存在，一些工作人员依法行政意识和主动服务意识还不强，落实能力有待进一步增强。对存在的问题，我们将首先从政府自身找原因、想办法，不掩饰、不回避，切实加以解决。

二、“十三五”时期的主要任务

“十三五”时期是我市全面建成小康社会的决胜时期，是全面深化改革的攻坚时期，是全面推进依法治市的关键时期。做好“十三五”时期各项工作，必须深刻领会、准确把握“五大发展”理念的基本内涵和实践要求，不断增强贯彻“五大发展”理念的自觉性坚定性，牢牢把握发展的主动权；必须深刻领会、准确把握习近平总书记关于经济发展新常态这一大逻辑的重要论述和具体要求，更加积极主动地适应和引领经济发展新常态；必须深刻领会、准确把握中央关于加强供给侧结构性改革的政策思路和重点任务，坚决打好供给侧结构性改革攻坚战；必须深刻领会、准确把握自治区党委、政府和市委关于“十三五”时期和2016年经济社会发展的目标要求，努力实现经济持续健康发展；必须深刻领会、准确把握中央和自治区党委、市委关于推进全面从严治党的部署要求，为打造祖国北疆亮丽风景线提供坚强有力保障。

在此基础上，我们要以高度的政治自觉和责任担当，率先发展，走在前列，努力在自治区调整优化产业结构、保持经济持续健康发展上起带头、作表率；在务实推进供给侧结构性改革、实现全面深化改革新突破上起带头、作表率；在加快推进城市基础设施建设、提高城市管理服务水平上起带头、作表率；在打好脱贫攻坚战、促进城乡统筹一体化发展上起带头、作表率；在落实全面依法治市要求、扎实推进平安首府建设上起带头、作表率；在全面落实从严治党政治责任、提升治政水平上起带头、作表率，切实发挥好首府在全区发展中的龙头带动作用。

按照上述要求和努力方向，今后五年全市主要发展目标是：经济发展实现“两个中高”；在全区率先实现“两个翻番”、“两个脱贫”；城乡面貌、人民生活、生态环境实现明显改善。

“两个中高”：一是经济保持中高速增长。主要指标增速保持在7%以上，其中地区生产总值年均增长7.5%，一般公共预算收入年均增长8%，固定资产投资年均增长10%，社会消费品零售总额年均增长8%，城乡常住居民人均可支配收入年均分别增长8%和9%。二是产业发展迈向中高端。新兴服务业占第三产业比重超过50%，先进制造业占工业比重超过20%，战略性新兴产业占工业比重超过40%，科技创新成为推动发展的新动力。三次产业比重调整为3∶27∶70。

“两个翻番”：地区生产总值到2019年达到3850亿元，较2010年末翻一番，2020年迈上4000亿元台阶；城乡常住居民人均可支配收入于2019年前分别达到50780元和19200元，较2010年末翻一番。这两方面指标率先在全区实现翻番。

“两个脱贫”：到2017年，全市农村建档立卡的25362人全部实现稳定脱贫，人均可支配收入达到4000元以上；清水河和武川两个贫困县全部摘帽。到2020年，贫困人口人均可支配收入提高到10000元以上。

“三个明显改善”：一是城乡面貌明显改善。完成地铁1、2号线、机场迁建等一批重点工程建设，城市空间布局合理，基础设施较为完善，品位特色、宜居水平和管理水平全面提高。常住人口城镇化率达到75%以上，户籍人口城镇化率达到60%以上，建成服务全区、辐射西北、对接京津冀、面向俄蒙、民族特色鲜明的区域中心城市。二是人民生活明显改善。全市人均GDP突破2万美元，居民消费支出占GDP比重达到30%以上，城乡居民收入达到全国中上水平，排在全区前列，基本实现“两个同步”，在全区率先全面建成小康社会。三是生态环境明显改善。二环路内实现“无煤化”，空气优良天数保持在90%以上；城市规划区内实现分质分类供水，城市污水应收尽收，排放标准优于一级A，再生水利用率提高到60%，城区水系碧波荡漾；城市建成区绿化覆盖率提高到40%，全市森林覆盖率提高到27.4%，宜居水平和可持续发展水平大幅提升。

围绕上述目标，“十三五”时期主要发展任务是：

(一)坚持创新发展，打造经济升级版。以鼓励引导大众创业、万众创新和文化创意为突破口，优化资源要素配置，促进新技术、新产业、新业态蓬勃发展，在全区率先实现动力转换。以供给侧结构性改革为着力点，围绕绿色食品加工、清洁能源、光伏材料、云计算等优势特色产业，打造一批国家级、世界级产业基地，推动产业向中高端迈进，在全区率先实现产业优化。以打造创新发展高地为目标，大力支持企业品牌建设和质量建设，培育和建设一批实验研究平台，突破和掌握一批关键核心技术，在全区率先形成发展新优势。以强化要素保障为支撑，发挥首府科教资源集中的优势，加快“高精尖”人才的培养和引入，夯实创新创意创业基础，在全区率先建成国家创新型城市。

(二)坚持协调发展，推进城乡发展一体化。以“生活空间宜居适度、生产空间集约高效、生态空间山清水秀”为发展方向，加快调整优化城市空间布局、产业布局和生态布局，提升可持续发展能力。以创造优良人居环境为目标，加快实施城市棚户区(城中村)改造，打通断头路，推进地下综合管廊、智慧城市建设，有效治理交通拥堵、环境污染等“城市病”，提升宜居水平。以补齐农村这块全面建成小康社会的短板为重要抓手，加快推进以人为核心的城镇化，全力实施好以“十个全覆盖”工程为重点的新农村建设，推动城镇公共服务向农村延伸，促进城乡公共资源均衡配置，实现城乡均衡发展。

(三)坚持绿色发展，建设人与人、人与自然和谐共处的美丽家园。更加注重生态建设，加快城市水系、绿带、小微绿地和城外河湖、森林、草原建设，构建完整的生态网络，打造城在林中、绿在城中、水系环绕、文明宜居的城市。更加注重节约资源，严格控制城市开发规模，科学划定城市边界，提升产业土地利用率，实现能源、资源的高效利用。更加注重低碳发展，大力发展循环经济和绿色产业，推进海绵城市和新能源城市建设，提高绿色经济比重。更加注重污染防治，深入实施大气、水、土壤污染防治行动计划，严格控制污染物排放，努力改善空气环境质量，提升污水收集处理率和再生水回用率，推进垃圾减量化、资源化，让首府的天更蓝、水更清、空气更清新。

(四)坚持开放发展，拓展发展新空间。努力扩大对外开放，主动融入国家“一带一路”倡议，积极参与中蒙俄经济走廊建设，全力办好中国-蒙古国博览会，加快建设综合保税区和中蒙自由贸易试验区，打造向北开放的“桥头堡”。努力强化区域合作，积极融入京津冀协同发展战略，加强与呼包银榆经济区和呼包鄂榆城市群的协作，加快建设连通区内外的机场、铁路和高速公路，巩固和提升区域中心城市地位。努力创新开放模式，大力发展互联网经济和跨境电子商务，在更高层次、更广范围构建开放型经济。努力扩大开放领域，全面实行准入前国民待遇和市场准入负面清单制度，积极有效引入国内外资金和先进技术，毫不动摇鼓励支持非公有制经济发展，促进各种所有制经济共同发展。

(五)坚持共享发展，推动发展成果全覆盖。更加注重对特定人群特殊困难的精准帮扶，全力打好脱贫攻坚战。坚持就业优先战略，多措并举满足劳动者就业创业需求。坚持“两个同步”，持续增加城乡居民收入。完善社会保障和救助制度，建成覆盖城乡居民、更加公平可持续的社会保障救助体系。加快发展教育事业，全力满足人民群众“上好学”的需求。深化医药卫生体制改革，实现人人享有基本医疗卫生服务和医疗保险全覆盖。深化文化体制改革，促进基层公共文化服务实现均等化，建设民族文化大市。加强和创新社会治理，创建平安首府，让人民群众有更多获得感、安全感和幸福感。

三、2016年重点工作

今年是全面建成小康社会决胜阶段的开局之年，是推进结构性改革的攻坚之年，也是做好迎接自治区成立70周年工作的关键之年。今年政府工作的总体要求是：全面贯彻党的十八大和十八届三中、四中、五中全会和中央经济工作会议精神，全面贯彻习近平总书记系列重要讲话和考察内蒙古重要讲话精神，全面落实自治区党委、政府和市委各项决策部署，坚持“五位一体”总体布局和“四个全面”战略布局，牢固树立和全面落实创新、协调、绿色、开放、共享发展理念，坚持稳中求进工作总基调，坚持稳增长、促改革、调结构、惠民生、防风险工作总要求，认真落实宏观政策要稳、产业政策要准、微观政策要活、改革政策要实、社会政策要托底的总体思路，按照“率先发展、走在前列”、“五个深刻领会、准确把握”和“六个起带头、作表率”的要求，着力在推进转型发展上下功夫，着力在推进供给侧结构性改革上下功夫，着力在去产能、去库存、去杠杆、降成本、补短板上下功夫，着力在加快推进以人为核心的城镇化上下功夫，着力在全面推进改革开放上下功夫，着力在为全市人民谋福祉上下功夫，努力实现“十三五”时期经济社会发展首年开门红。

全市经济社会发展主要预期目标是：地区生产总值增长7.5%，一般公共预算收入增长8%，规模以上工业增加值增长9%，固定资产投资增长12%，社会消费品零售总额增长8.5%，城乡常住居民人均可支配收入分别增长8%和9%，居民消费价格涨幅控制在3%以内。

今年，我们要紧紧抓住受到国务院督查表扬并给予多项激励措施的重大机遇，再接再厉、奋勇争先，全力做好以下工作。

(一)加强供给侧结构性改革，促进经济持续健康发展

全面落实供给侧结构性改革五大任务。因地制宜化解落后产能。以消化过剩产能、淘汰落后产能为重点，研究制定总体实施方案和分类推进措施，实现去产能与促转型协调推进。通过严格环保、安全、能耗标准，推进企业兼并重组、破产清算，积极消化尿素、电解铝、水泥等领域过剩产能。通过实施“互联网+”行动计划及技术改造，强化财政、税收、金融政策保障，推动传统行业、困难企业调整结构、加快转型、拓展市场。扎实有效消化房地产库存。出台购买商品房减税减费政策，适当放宽住房公积金发放条件，培育释放刚性和改善性住房需求；全面推进棚户区(城中村)改造货币化安置，回迁购买一批；激活住房租赁市场，发展创客地产、“三创”社区搞活一批；引导鼓励企业以市场为导向调整商品房户型结构，开发养老、文化、体育、科教等新型房地产业，全力打通供需通道，转型升级一批；积极推进户籍制度改革和住房制度改革，鼓励农民进城，就业购买一批；加快解决环卫工人、公益性岗位从业者等相对困难群体住房问题，政策保障一批，有效缓解房地产库存压力。多措并举降低企业成本。扩大大用户直购电范围和电力多边交易市场，切实降低主导产业用电价格和成本。发挥好再生水的低成本优势，提高化工、电力行业竞争能力和公共服务水平，把降成本、去产能和发展新兴产业、公共服务行业有机结合起来。积极推进流通体制改革，完善三级物流配送网络，科学布局大型物流园区、分拨配送中心和末端网点及物流信息服务平台建设，大力发展新型便捷的物流配送模式，构建更加高效快捷的物流配送体系。千方百计补齐发展短板。以“十个全覆盖”、脱贫攻坚工程为抓手，加快推进基本公共服务向乡村延伸。加强政策引导和金融支持，促进发展要素向传统产业改造升级集中，向战略性新兴产业集聚，加快推进产业转型升级。以地下综合管廊、机场迁建、地铁、道路交通、环保整治、生态绿化为载体，扩大有效投资，全面提升基础设施保障和服务水平。全力以赴稳控金融风险。推进全口径政府债务管理，做好政府存量债置换工作，有效化解政府债务风险。认真梳理政府与企业债务关系，扎实做好债务清偿、债权清缴工作，切实保障各类有效投资的合理流动性。强化金融监管，规范各类融资行为，坚决遏制和打击非法集资。加强风险监测预警，妥善处理涉险案件，坚决守住不发生区域金融风险的底线。

着力培育创新环境。强化科技创新引领作用。设立创新奖励扶持资金，围绕重点领域重点行业，加快实施关键技术攻关、实用成果转化、人才培育引进、平台载体建设，年内完成呼和浩特科技创业服务中心建设，培育10家以上国家级高新技术企业，新增后备上市高新技术企业5家。深化高校、科研院所与政府和企业的沟通合作，启动呼和浩特公共实训中心项目，推动重点企业联合高校、科研院所组建研发机构10家以上。推进大众创业、万众创新和文化创意，年内争取5个众创空间试点基地获得自治区认定，集中扶持回民区金海网创、玉泉区大盛魁文创、赛罕区农创、开发区留创等园区和新城区“三创”社区建设，打造众创、众包、众扶、众筹服务平台。加快发展互联网经济。大力实施《中国制造2025》国家战略和“互联网+”行动计划，加快现代信息技术与制造业、服务业、农牧业的深度融合。支持伊利、蒙牛等企业完善大数据共享平台建设，全面实现管理和产品数字化；推动中环光伏实施工业4.0智能化改造，实现产能和功能同步升级；加快传统产业信息化、智能化改造，实现生产用能全过程动态监控和优化管理；结合“宽带中国”示范城市建设，鼓励支持基于大数据和互联网的商贸物流配送体验，推动线上线下就地交易。

加快推动重点产业和基础设施项目建设。组织实施一批打基础、利长远的重大工程，新建续建亿元以上(房地产5亿元以上)项目371个，其中，产业项目68个，基础设施项目11个，城镇化项目209个，生态环境和节能减排项目23个，社会民生项目52个，农林水项目8个，全年力争完成投资1056亿元。完成白塔机场迁建报批并正式开工，加快建设呼张高铁、呼准鄂城际铁路，开工建设老牛湾通用机场。全面启动国道209线呼武段大青山隧道及引线工程、国道110线呼协段改造工程、清水河冯家塔至老牛湾沿黄旅游公路建设，积极推进省道311线武川至杨树坝公路建设前期工作，完成呼塔一级公路二期工程。加快实施一批水利、电力改造建设项目，切实提升城乡发展保障能力。

(二)大力发展现代都市农业，加快补齐农村全面小康短板

按照“绿色安全、服务城市”的定位，突出抓好三类产品有效供给。继续鼓励土左旗、托县、和林县等具备条件的地区大力发展奶牛规模化养殖、优质牧草种植、设施蔬菜等产业，提供优质安全的农畜产品。年内使本地蔬菜供应率提升5个百分点，奶牛单产提高1吨，牧草亩产增加200公斤。大力开发旅游休闲观光产品，在市四区城郊地区培育发展一批高端农创园、采摘园、观赏园、农耕文化园，打造集观光、休闲、体验、美食为一体的农家休闲旅游服务体系，提高农业综合效益，推动农林牧渔结合、一二三产业融合发展。着力培育山水田园、生态绿色产品，加大对森林、湖泊、草原、湿地的保护力度，制定切实可行的保护性开发规划，守住生态红线。重点支持武川县、清水河县等丘陵草原地带在推进移民搬迁的同时，大力发展花草、林果等产业，实现生态与生活共美共赢。

全力打好“十个全覆盖”工程攻坚战，深入推进美丽乡村建设。按照“以人为本、农民主体，保质保量、厉行节约”的原则，投入60亿元以上，对剩余1368个行政村及自然村实施“十个全覆盖”工程，对1170个村庄实施移民搬迁工程，3月底全面启动，9月底基本完工。加大传统村落、民居、历史文化名村名镇保护力度，打造一批具有历史记忆、地域特点、民族风情的美丽宜居乡村。加大村容村貌管理投入，积极开展人居环境综合整治，加快形成“政府引导、社会支持、农民参与、共建共享”的长效管理机制，让乡风文明、村容整洁成为农村发展常态。

全力推进脱贫攻坚计划，提高贫困农民生活水平。坚持精准扶贫、精准脱贫，按照贫困地区和贫困人口的具体情况，深入实施“五个一批”工程。重点实施产业脱贫，选择一批具备产业发展条件和贫困人口相对集中的村实施整村推进。结合易地搬迁、生态恢复工程，通过转移就业和保护生态安置帮助贫困农民实现脱贫。落实金融扶贫富民工程，积极为有发展意愿和劳动能力的困难群众解决资金难题。继续加大教育扶助力度，扩大社会保障覆盖范围，提高医疗救助水平，全年确保14890人实现精准稳定脱贫。

全面深化农村综合改革，激发农村发展活力。加快培育新型职业农民队伍和农业经营主体带头人，引导农村青年、返乡农民工、农村大中专毕业生等加入职业农民队伍。完善农业产业链与农民的利益联结机制，发展订单农业，引导农户以土地经营权入股龙头企业和专业合作社，让农民真正融入现代农业产业链条，获得合理的产业链增值收益。深化农村集体产权制度改革，依法开展土地经营权有序流转，扎实推进和林县农村土地征收改革试点工作，健全农业农村投入持续增长机制。推进农业标准化和信息化。做好全国第三次农业普查工作。

(三)推动工业转型升级，增强经济发展动力

加快发展战略性新兴产业。开工建设碳谷科技富勒烯碳纳米新材料、山东天岳碳化硅晶体材料、内蒙古矿业集团稀贵金属材料、深圳沃特玛新能源汽车、亚太锅炉压力容器等新兴产业项目，推动新材料、新能源汽车、现代装备制造等产业做大做强。争取庆华煤基清洁能源多联产、锦江粉煤灰综合利用项目获得核准，中环单晶硅四期、晶环蓝宝石三期等项目开工建设，科林埃尔3GW电子材料产业园投产运行，进一步提升新型煤化工、光伏产业发展水平。用足用好新兴产业差别化支持政策，发挥好创投基金引导作用，以特色产业园区为平台，努力争取一批新的重大项目落地建设。

积极推进传统产业改造升级。认真落实国家、自治区优惠政策，支持伊利、蒙牛等食品加工企业加快技改、研发进程，深化全球发展战略，加快圣牧高科等低温乳制品加工基地、正大集团等肉制品加工基地建设。积极推动生物发酵产业整体转型、成品药升级换代工程，今年确保金宇国际生物科技产业园建成投产，阜丰科技氨基酸生产线绿色升级技改、常盛制药产品升级等项目全面完工。加快北方联合电力和林电厂、京能盛乐热电、大唐托电五期等重大电力项目建设进度，启动建设一批风力发电、光伏发电项目，推动清洁能源输出示范基地建设取得重要进展。

大力扶持中小微企业发展。实施中小微企业创新发展工程，鼓励企业通过技术创新、品牌创建做大做强。引导传统中小企业立足优势特色产业做好协作配套，实现提质增效、转型升级。新建2个中小微企业公共服务平台，构建更加完善的社会化服务体系。建设中小微企业产业园，引导企业集中集聚发展。认真落实“助保贷”等融资服务政策，积极扶持中小微企业上市融资或在“新三板”挂牌，着力解决企业融资难题。

(四)加快发展现代服务业，培育转型发展新动能

深入推进国家服务业综合改革试点和自治区服务业改革创新试点工作，强力推进服务业发展专项行动，推动生产性服务业向专业化和价值链高端延伸，生活性服务业向精细化和高品质转变。

做大做强生产性服务业。开展云计算产业应用行动。年内新增15万台服务器、20万个互联网接入点，开通5个云数据端口；全面推进智慧城市建设，积极推广政务云应用，推动全市视频监控等数据资源有效整合和共管共享；大力实施信息惠民工程，加快云计算应用示范中心、国家新媒体产业园等项目建设，培育打造更加完善的云计算产业链条。开展金融总部服务业创新行动。积极引进大型商业银行以及证券、保险、期货、信托等金融机构，力争国家进出口银行、平安银行入驻我市；深化与平安、中信、太平保险等金融机构合作，促成总规模400亿元的城市发展、交通产业投资、美丽乡村建设等3支基金落地；规范发展互联网金融，争取2家民营网络银行获批建设；研究出台支持总部经济加快发展的政策意见，不断壮大以如意总部基地为核心的总部集聚区。开展现代物流业提升行动。加快沙良铁路公路物流园区后续建设，推动沙尔营物流园区完成战略转型，筹备建设呼和浩特铁路口岸；争取天津“无水港”落户我市，重新开通“如意号”跨境货运班列，培育建设辐射内陆、连通欧亚的跨境物流大通道；规范发展快递配送服务，合理布局城乡配送网点，切实解决物流配送成本高、管理差和最后一公里问题。开展会展经济品牌行动。创新会展业运行机制，围绕优势产业发展、特色产品生产销售，积极培育草原民族特色展会、消费类展会、网络展会、《中国制造2025》展会等专业品牌，年内争取举办各类展会100场以上，成交额及意向协议总金额超过120亿元。

做精做优生活性服务业。开展商贸服务业转型行动。大力支持传统商贸企业搭建电子商务交易平台，鼓励发展线上贸易，力争线上贸易额占交易额20%以上，培育打造本土特色电商品牌和体验店；加快推进电子商务园区建设，促进电子商务集中集聚发展；加快发展跨境电子商务，进一步畅通国际快件通道，创建国家跨境电子商务综合试验区；培育农村电商服务平台，积极发展农村电子商务。开展文化旅游产业融合升级行动。围绕文化+科技、文化+历史、文化+旅游、文化+会展“四大板块”，加快总投资600多亿元的23个重点文化旅游产业项目建设。依托科技深度融合，积极引进培育一批在全区全国有较高影响力、较强实力的文化园区和企业，重点实施好华夏文明传承文化产业园、蒙元世界草原文化产业园、内蒙古影视文化产业科技园等项目建设。依托积淀丰富的历史文化资源，大力实施一批体现草原文化、丝路文化的文化旅游产业项目，重点推进大盛魁文化创意产业园、呼和浩特草原马汇等项目建设。依托丰富的自然风光资源，着力建设一批具有地区影响力的精品文化旅游项目，重点加快老牛湾黄河峡谷旅游区、敕勒川文化旅游产业园、神泉生态旅游区等项目建设。依托中国—蒙古国博览会与“一会两节”等会展节庆，全力打造一批商贸会展及文艺精品项目，重点办好第十七届昭君文化节、第六届少数民族文化旅游艺术活动和第二十七届自治区旅游那达慕大会。深入开展“旅游厕所革命”行动，实施智慧景区建设计划，提升旅游服务水平。加强旅游推广宣传，推动区域旅游一体化发展，提高首府旅游知名度。开展体育产业振兴行动。深入推进足球运动改革发展，加快专业化体育场馆建设和老旧场馆改造，积极引进专业赛马、电动方程式赛车、国际足球、冰雪运动等大型体育项目，培育打造一批具有显著影响力的体育赛事，带动群众性体育事业全面发展。开展健康养老生活服务业培育行动。加快推进日间照护中心、托老所、互助式养老服务中心和农村养老幸福大院等养老服务设施建设，实施市第一医院、中蒙医院2个医养结合项目，新增养老床位2700张，加快构建更加完善的养老服务体系。启动建设全市统一的家庭服务网络平台，打造更加便利、规范的家庭服务体系。

(五)坚持人民城市为人民、文明城市人民建，提高共建共管共享水平

加快重大基础设施建设。全面开工建设地铁1、2号线，同步启动新华广场和火车站广场改造工程，完成快速路后续配套工程及BRT快速公交站点建设。新建改造供排水及再生水管网300公里、供热管网421公里(包括二次供热管网)、燃气管网111公里，完成察哈尔大街、科尔沁北路24公里综合管廊建设，着力补齐城市地下基础设施短板。新建扩建220千伏变电站4座，新增变电容量1200兆伏安，增强用电安全稳定性。完成儿童探索博物馆、规划馆、自治区美术馆、殡仪馆、少数民族群众文化体育运动中心主体工程建设，加快推进国家(北方)足球训练中心、公共资源交易中心建设，着力完善城市服务功能，提升公共服务能力。

下大力解决拥堵问题。以“疏”为主，完善城市道路体系。新建续建道路、桥梁和通道38项，过街天桥21座，改造连接路90条，打通断头路65条，改造维修小街巷19条，对中心城区拥堵路段实施拆迁拓宽改造，提升道路通行能力。以“严”为主，强化城市道路交通管理。严格规范路边停车，启动建设公共停车场30个、立体停车场5个，在34条小街巷实施交通单行，打通道路微循环系统。以“便捷”为主，推进“公交都市”建设。开工建设2座公交综合场站，新增新能源公交车327台，新建充电桩41个，规划设置公交专用车道50公里，提高公交出行率和保障服务水平。

提升城市宜居水平。深入推进城市拆改建三年行动计划，对35个棚户区(城中村)、138处城市边死角进行改造，完成拆迁700万平方米以上。整治改造老旧小区284个、466万平方米，同步对75个片区、296个老旧小区供排水管网和二次加压设备进行改造，对1554个老旧小区实施准物业长效管理，实现水、电、气、暖等市政管理服务延伸入户。启动直饮水工程。新建续建便民市场130个。对新华西街、海拉尔西街、通道南北路、腾飞路、机场路等主要街道实施街景整治。完成草原丝绸之路主题公园建设，新建改建公园游园8个，新增绿地5000亩，为市民打造更多休闲运动场所。

加强城市精细化管理，完善城乡规划编制，实施“多规合一”。编制城乡全域总体规划，修编完善中心城区控制性详细规划，做好重点区域城市设计，使城乡建设有“规”可依、有“章”可循。深入实施市容环境三年综合整治行动，全面清理整治占道经营、乱倒渣土、随意开挖道路、车窗抛物、私搭乱建等行为。持续增加对环卫设施的投入，启动建设各类环卫场站14座，改造垃圾转运站50座，购置各类环卫车辆200台，将金川以东、大青山以南、机场河西路以西、大黑河以北范围内城中村全部纳入城市一体化管理范围。继续保持打击“两违”的高压态势，实现违建项目“零增长”、新建项目“零违章”。完善土地储备制度，依法查收闲置土地。增强广大市民卫生意识、文明意识、城市意识，形成共建、共管、共享的城市发展氛围，积极创建国家卫生城市、国家生态园林城市和全国文明城市。

加快小城镇建设。按照“小而美、小而特、小而强”的要求，加快重点集镇改造建设，着力提升基础设施建设水平、公共服务保障能力、吸纳就业及辐射带动能力，打开以工促农、以城带乡的现实通道。

(六)改善生态环境，增强可持续发展能力

加快治理大气污染。着力构建以热电联产为主、清洁能源为补充的供热保障体系，完成京能盛乐电厂热源入呼工程，对现有供热机组实施挖潜改造，加快淘汰分散及小型燃煤锅炉，对65蒸吨以上供暖及生产性企业燃煤锅炉进行脱硫脱硝改造，积极推进近郊村庄集中供热或清洁能源供热，切实减少排放污染。推广使用新能源汽车，控制和治理汽车尾气排放。强化建筑工地、道路运输扬尘管理，对城区裸露的渣土堆进行整治，建设小微绿地和公园。

全面治理水污染。完成城区污水处理厂扩容和提标工程，推进雨污水分流工程，新增污水收集管网83公里，城市污水收集率和处理率提高到95%，排放标准优于一级A。加快建设再生水管网，城市再生水利用率由20%提高到35%。启动“五河两库”治理工程，大黑河城区段10月份实现碧波荡漾。严格控制地下水开采，封闭公共供水管网覆盖范围的自备井130眼。启动海绵城市建设，实施雨水自然积存、渗透、净化项目，高效合理利用水资源。

加大生态建设和保护力度。全面完成国家和自治区重大生态修复工程建设任务，实施好京津风沙源治理、天然林保护、退耕还林等重大生态修复工程，完成绿化造林20万亩。实施65公里长的城市快速路园林绿化、101公里长的绕城高速宽林带绿化，巩固提升44公里长的环城水系景观和六个出城口生态景观，完成大青山前坡生态休闲观光带和14.3公里长的大黑河生态绿化带建设，打造“三环两带”五道亮丽的生态绿化景观带。进一步提升乌素图、哈达门国家森林公园建设水平，加大哈素海湿地、黄河湿地、大青山北坡保护建设力度，促进生态与经济协调发展。

(七)加大改革攻坚力度，进一步提高开放水平

推进改革举措落地生根。全面实行并联审批、联审联办制度，持续深化简政放权、放管结合、优化服务改革。打造“区市合一”公共资源交易平台。以市场为导向，加快推进国有企业分类改革和薪酬制度改革，整合粮食、供热、市政工程等同类市属国有企业。完善国有资产管理体制，以管资本为主加强国有企业资产监管。加快推进企业登记注册便利化，实现电子营业执照和企业注册全程电子化。积极推进不动产登记工作。做好和林县国家新型城镇化综合试点工作。继续深化财税体制改革，完善政府和社会资本合作模式，创新项目投资和分配机制，扩大PPP项目实施规模，持续增加有效投资。推行优先使用创新产品、绿色产品的政府采购政策。

全面扩大对外合作。积极推进中蒙俄经济走廊建设，启动建设中国-蒙古国博览会永久会址，全力争取中蒙自由贸易试验区获批。以现有的货运包机航线和国际快件通道为支撑，以海悦通、恒鑫源、俄蒙通等项目为抓手，积极推动呼和浩特出口加工区转型升级为综合保税区。充分利用我市区位、交通、产业基础优势，从扩大清洁能源供给、承接产业转移、提供休闲度假服务等合作内容入手，深化与环渤海、珠三角、长三角等沿海发达地区的合作。进一步强化呼包银榆经济合作区建设，推动呼包鄂协同发展，全力打造以我市为中心的区域性合作平台。

(八)加快社会事业发展，健全完善公共服务体系

坚持优先发展教育。实施好学前教育三年行动计划。加大义务教育投入，推进城乡义务教育办学条件标准化，年内新建续建中小学、幼儿园24所，完成4个县区国家义务教育发展基本均衡县评估认定。深化考试和招生制度改革，完善中考招生方案。公开招聘一批教师，加强教师队伍特别是乡村教师队伍建设。完善现代职业教育体系建设。以义务教育和职业教育为重点，加快发展民族教育。进一步提高民办教育、特殊教育办学水平。加快智慧校园建设，深入推进教育信息化。

推进健康呼和浩特建设。以“三医联动”为推手，加快城市公立医院综合改革，抓好分级诊疗试点和医师多点执业。完成市中蒙医院、妇幼保健院和284个标准化村卫生室建设，启动市第二医院迁建工作。组建医疗集团或医疗联合体，加强诊疗合作交流。健全完善药品供应保障体系，减轻群众就医负担。全面落实乡村医生养老退养政策。鼓励社会力量办医。扶持蒙中医药事业发展。完善人口经济发展战略，全面落实一对夫妇可生育两个孩子政策。

推动文化事业繁荣发展。坚持社会主义先进文化前进方向，用中国梦和社会主义核心价值观凝聚共识、汇聚力量。加强思想道德和社会诚信体系建设，增强国家意识、法治意识、社会责任意识，倡导科学精神，弘扬中华传统美德。加大文化遗产保护力度，实施好昭君博物院、将军衙署、市博物馆、乌兰夫纪念馆等改造提升工程，推出2-3部舞台艺术精品剧目。深化文化体制改革，完善公共文化服务体系，创建国家公共文化服务体系示范区。

全面加强社区建设，增强服务功能，稳定工作队伍。注重发挥工会、共青团、妇联等群团组织的桥梁纽带作用，大力支持老龄、残疾人、红十字和慈善事业，深入实施呼和浩特市妇女儿童发展纲要(2011-2020年)，扎实做好统计、档案、气象、地震、外事侨务等工作，推进各项社会事业协同发展。

(九)着力保障和改善民生，维护社会和谐稳定

实施更加积极的就业创业政策。统筹做好高校毕业生、农村剩余劳动力、城镇就业困难人员的就业工作。加大创业扶持力度，完成大学生创业就业综合服务中心、首府人才市场改造和创业就业服务平台建设。实施新生代农民工职业技能提升计划，组织引导劳务输出，促进农村剩余劳动力转移就业。做好困难群体就业，确保零就业家庭动态清零。

建立更加公平更可持续的社会保障制度。实施全民参保计划，进一步扩大社会保险覆盖面。有序推进养老保险制度改革，落实“五险一金”精简归并政策。适当提高大病保险人均筹资水平和支付比例。进一步完善机关事业单位工资制度。加大社会保险费征缴力度，着力解决众环集团、公交公司等25家国有企业到龄退休人员的养老待遇问题。健全完善城乡社会救助体系。继续落实好“三个一”民生实事。完善城乡低保户、特困人员相关救助政策，保障好困难群众的基本生活。今年农村低保标准提高100元/年·人，农村五保集中供养标准提高700元/年·人。

维护社会和谐稳定。深入开展平安首府建设，推进网上信访和联合接访机制建设，着力解决好回迁房安置、房屋产权证办理、农民工工资拖欠等信访问题。加强公安派出所基础设施建设，以信息化为支撑加快建设社会治安立体防控体系，严密防范、依法惩治违法犯罪活动。加强安全生产监管，落实安全生产责任，抓好燃气、消防、交通、建筑和特种设备等重点领域隐患排查和专项整治，坚决防止重特大事故发生。加强食品药品安全监管能力建设，争创国家食品安全城市。积极支持国防和军队建设，提升军民融合发展水平。全面落实党的民族政策和宗教工作基本方针，深入持久开展民族团结进步创建活动，巩固和维护好首府安定团结的良好局面。

(十)切实加强自身建设，打造人民满意政府

严格遵守党的政治纪律和政治规矩，不断强化政治意识、大局意识、核心意识、看齐意识，坚定自觉地同以习近平同志为核心的党中央保持高度一致。坚决贯彻落实党中央国务院、自治区党委政府和市委各项决策部署，增强政府执行力。自觉接受市人大及其常委会的法律和工作监督，主动向人大及其常委会报告工作，坚决执行人大的各项决议和决定，虚心接受人大的工作评议，切实提高依法履职尽责能力。主动接受市政协的民主监督，搞好协商议政，定期通报重大决策部署和重要工作落实情况。充分听取人大代表、政协委员、民主党派、工商联、无党派人士和人民团体的意见建议，认真组织实施政府协商计划，积极推进决策科学化民主化。

加强法治政府建设，严格按照法定权限、法定程序和权力清单、责任清单履职尽责，将政府活动全面纳入法治轨道。健全完善地方法律法规体系，修改5件政府规章和2件规范性文件，提请市人大常委会制定5件地方性法规。加强法制教育宣传，深入开展“七五”普法，弘扬社会主义法治精神，增强全民法治观念，在全社会形成良好法治氛围和法治习惯。

持之以恒改进工作作风，深入开展“勇于担当、攻坚克难”主题活动，提升服务水平，增强干事创业热情。落实从严治党要求，加强政府系统党风廉政建设，认真学习贯彻《中国共产党廉洁自律准则》、《中国共产党纪律处分条例》，坚决纠正和惩处不作为乱作为、懒政怠政、失职渎职行为。加强对重点项目推进、重大资金使用的专项审计，建立扎实有效的自查自纠机制。完善惩治和预防腐败体系，巩固反腐败成果，使不敢腐的震慑作用充分发挥，不能腐、不想腐的效应进一步显现。

各位代表，“十三五”发展征程已经开启，全面建成小康社会的冲锋号已经吹响，让我们更加紧密地团结在以习近平同志为核心的党中央周围，在自治区党委、政府和市委的坚强领导下，紧紧依靠全市各族人民，守望相助，团结奋斗，锐意进取，共同开创首府改革开放和现代化建设的新局面!
